# Design of a Completely Vertical, Polarization-Independent Two-Dimensional Grating Coupler with High Coupling Efficiency

**DOI:** 10.3390/s23104662

**Published:** 2023-05-11

**Authors:** Chung-Chih Lin, Yen-Cheng Lu, Yu-Hsuan Liu, Likarn Wang, Neil Na

**Affiliations:** 1 Institute of Photonics Technologies, National Tsing Hua University, Hsinchu 30013, Taiwan; 2 Artilux Inc., Hsinchu 30288, Taiwan

**Keywords:** CMOS compatibility, silicon photonics, grating coupler, completely vertical coupling, polarization-independent coupling

## Abstract

An efficient optical coupler to transfer the signal between an optical fiber and a silicon waveguide is essential for realizing the applications of silicon photonic integrated circuits such as optical communication and optical sensing. In this paper, we numerically demonstrate a two-dimensional grating coupler based on a silicon-on-insulator platform to obtain completely vertical and polarization-independent couplings, which potentially ease the difficulty of packaging and measurement of photonic integrated circuits. To mitigate the coupling loss induced by the second-order diffraction, two corner mirrors are respectively placed at the two orthogonal ends of the two-dimensional grating coupler to create appropriate interference conditions. Partial single-etch is assumed to form an asymmetric grating to obtain high directionalities without a bottom mirror. The two-dimensional grating coupler is optimized and verified with finite-difference time-domain simulations, achieving a high coupling efficiency of −1.53 dB and a low polarization-dependent loss of 0.015 dB when coupling to a standard single-mode fiber at approximately 1310 nm wavelength.

## 1. Introduction

Silicon-on-insulator (SOI) structure provides a promising platform for implementing photonic integrated circuits (PICs) due to the outstanding optical confinement arising from its high refractive index contrast. Features such as low optical loss, high integration density, and CMOS compatibility make it possible to integrate various optical components into a single SOI chip. The coupling of optical signals with optical transceivers and sensors is of paramount importance in the practical implementation of PICs. However, due to the large size mismatch between the optical fiber mode (approximately ten micrometers) and the Si waveguide mode (approximately sub-micrometer), how to efficiently couple the signal between the optical fiber and the Si waveguide becomes a crucial issue to make PIC practical. Enlarging the mode size at the input end of a waveguide [1,2] or using a lensed fiber to reduce the incident spot size [3] is a straightforward solution to mitigate the coupling loss arising from the mode mismatch. Edge couplers employ an inverse tapered waveguide to enlarge the mode size at the input end for coupling and exhibit a high coupling efficiency and a low polarization-dependent loss (PDL) [2]. However, then an additional polish step is required to obtain a smooth cleaved facet that reduces the optical loss caused by surface roughness scattering. This limits mass manufacturability and renders wafer-level testing impossible, henceforth increasing the cost of the packaging dramatically. Lensed fibers can focus the incident light into a sub-micrometer spot and couple into the Si waveguide, but the requirement of high precision alignment in all three dimensions increases the difficulty in obtaining efficient coupling, henceforth again increasing the cost of the packaging dramatically. On the other hand, grating couplers fabricated on the top of the wafer surface may be a more attractive solution. Unlike edge couplers or lensed fibers, the polish-free grating coupler enables wafer-level testing and therefore significantly reduces the cost of testing. Moreover, the grating area can be easily fabricated to be as large as the dimensions of the fiber core, and nearly perfect mode matching can be achieved through optimizing the grating pattern [4], which all leads to better alignment tolerance and circumvent the use of an edge coupler or a lensed fiber.

There are various types of grating couplers designed to optimize different performance parameters, including the applications of a distributed Bragg reflector (DBR) or a metal mirror under the grating [5,6,7], an asymmetric structure [8,9,10,11], and an apodized grating [4,12,13,14]. A bottom mirror can be added to recycle the downward waves and increase directionality, but the processes needed often complicate the fabrication [5,6,7]. An asymmetric structure providing constructive/destructive interference among upward/downward waves improves directionality, but if a dual-etch process is applied, it suffers from the risk of exposure misalignment, which degrades the optical performance [9]. An apodized grating is commonly utilized to offer a Gaussian power distribution to match the fundamental mode of a standard single-mode fiber, but such an approach requires intensive computing resources to simulate and implement [12,13]. Nevertheless, with the advancement of these techniques, progressive developments of one-dimensional (1D) and two-dimensional (2D) grating couplers have been made over the past years. The 1D grating coupler design with high coupling efficiency near unity has been achieved [15], but its polarization-dependent coupling inherently limits its application as an optical receiver. In this regard, a 2D grating coupler should be a better solution as it accepts external optical fields with arbitrary states of polarization. However, it suffers from second-order diffraction if the incident angle is completely vertical. Consequently, many 2D grating coupler designs apply off-normal incidence to avoid the second-order diffraction problem, but then the problem of PDL occurs and the grating pattern is much more difficult to design. Moreover, in the case of off-normal incidence, the alignment between the optical fiber and the grating coupler becomes a three-dimensional (3D) problem, making both the testing and packaging much more complicated compared to a 2D problem in the case of normal incidence.

**Figure 1:**
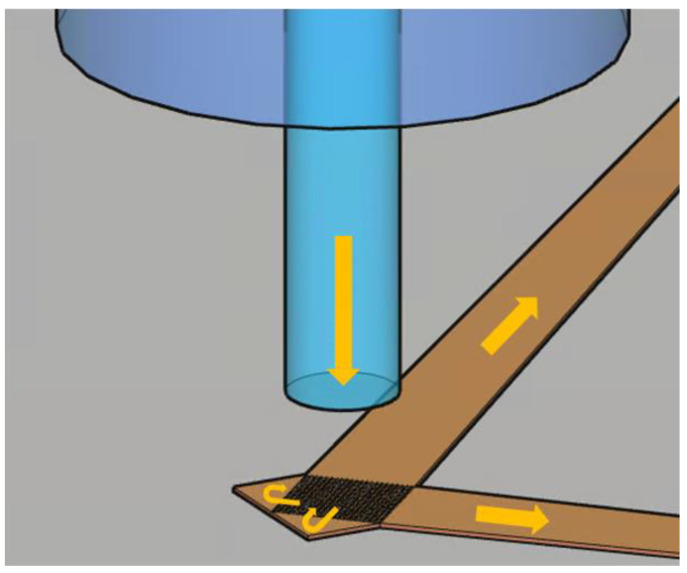
Schematic diagram of the 2D grating coupler as an optical receiver. The optical signal with two orthogonal polarizations from an external optical fiber is incident on the 2D grating coupler and is then converted to the two TE modes of the two Si tapered waveguides, respectively. The corner mirrors at the end of the gratings reflect the coupled incident light back to the Si tapered waveguides.

To enable normal incidence, the impact of the second-order diffraction must be overcome, and when the grating coupler is configured as an optical transmitter, the concept of imposing destructive interference to the second-order diffraction-induced back-reflection through simple reflection or critical coupling [16,17] has been proposed. In this paper, we extend our previous work on a 1D grating coupler [17] to a 2D grating coupler, by considering two corner mirrors respectively placed at the two orthogonal ends of the 2D grating coupler containing hole or pillar arrays in a square-lattice configuration. Figure 1 shows the schematic diagram of this 2D grating coupler as an optical receiver, where an asymmetric grating is formed by partial single-etch to increase the directionality. The hole depth or pillar height is fine-tuned to obtain high directionality, and the position of the corner mirror is also fine-tuned to obtain the required interference condition that utilizes second-order diffraction [17]. This structure is optimized and verified by finite-difference time-domain (FDTD) simulations, in which a single simulation unit cell is utilized for optimizing most structural parameters, and then a full simulation structure is utilized for verifying some key performance indices. Finally, we report a design in which a coupling loss of only 1.53 dB and a PDL below 0.02 dB can be achieved.

## 2. Design and Unit-Cell Simulation

**Figure 2:**
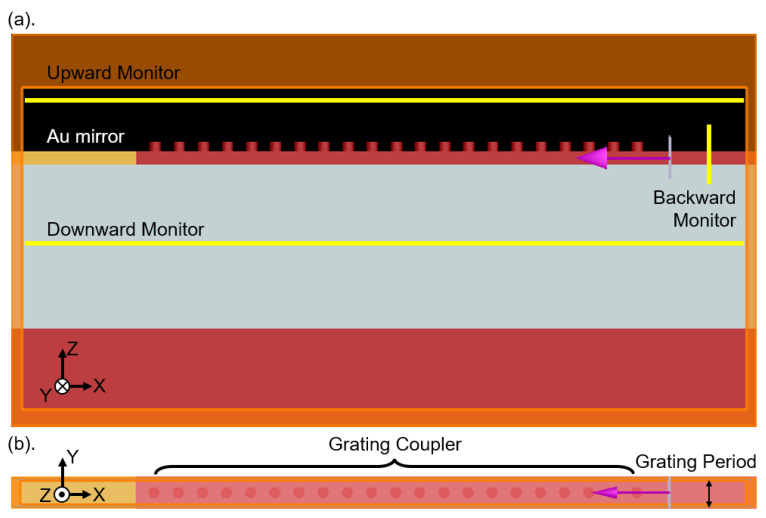
The unit-cell structure of the pillar array case used to simplify the simulation. (**a**) Side view. (**b**) Top view. The boundary condition in the y-direction is set to be periodic, and a virtual Au mirror replaces the actual corner mirror to reduce the simulated length in the x-direction. The fundamental TE mode of the Si waveguide is launched and propagates to the grating coupler, and power monitors are placed around the grating coupler to calculate the power distributions.

The 2D grating coupler under study is based on an SOI platform with a 250 nm-thick top Si layer and a 3 μm-thick buried oxide layer. It consists of a 2D grating, which can be treated as two orthogonally overlapped 1D gratings, two tapered Si waveguides connected to the 2D grating to guide the TE-polarized light incident on the grating region to either one of the two tapered Si waveguides and two corner mirrors respectively placed at the two orthogonal ends of the 2D grating coupler. The grating region consists of arrays of holes or pillars in square-lattice configuration with a side length of approximately 10 μm, which is designed to match the standard core size of SMF-28 optical fiber. We use the commercial FDTD simulation software developed by Ansys/Lumerical to optimize this structure. Many parameters are involved in the simulations, including the grating period, duty cycle, corner mirror position (referred to the as corner mirror shift in the following), and hole depth or pillar height. Thus, in principle, massive computations are necessary for the 3D simulation of the entire structure. However, due to the structural symmetry resulting from the intended completely vertical coupling, it suffices (1) to use only one of the polarization directions in the simulation, because this 2D grating coupler can be considered as two identical 1D grating couplers overlapped orthogonally; (2) to apply periodic boundary condition in the direction perpendicular to the waveguiding direction, with one row of holes or pillars as the unit-cell due to the existence of discrete translational symmetry; and (3) to replace the actual corner mirror with a virtual Au mirror for further simplification, where the optical path needed for the actual corner mirror will be identified in later full structure simulations. Figure 2 shows the resultant schematic diagram of the simulated unit-cell structure, which significantly reduces the loading of computations and speeds up the sweeping of parameters (the simulation time for each run of a unit-cell simulation and a full structure simulation are approximately 10 min and 10 h, respectively). In the following simulations of the unit-cell structure, we launch a fundamental TE mode of the Si waveguide into the grating coupler, with power monitors placed at the upward, downward, and backward positions, respectively, to monitor the power distributions in the configuration of chip-to-fiber coupling.

**Figure 3:**
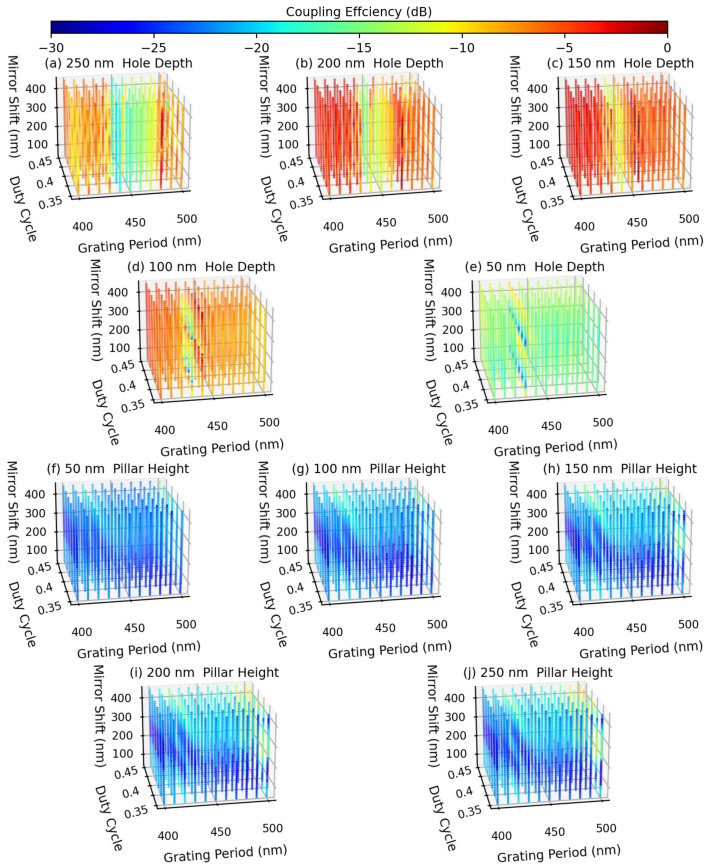
The relative coupling efficiency at 1310 nm wavelength is simulated for the unit-cell structure shown in Figure 2, given the Si waveguide thickness ranging from (**a**) 250 nm to (**e**) 50 nm when adopting the hole depth geometry, and from (**f**) 50 nm to (**j**) 250 nm when adopting the pillar height geometry. The grating period ranges from 400 nm to 500 nm, the duty cycle ranges from 0.35 to 0.45, the Au mirror shift ranges from 50 nm to 450 nm, and the grating hole depth/pillar height ranges from 50 nm to 250 nm. Different colors in the figures follow the color chart (in units of dB) on top.

**Figure 4:**
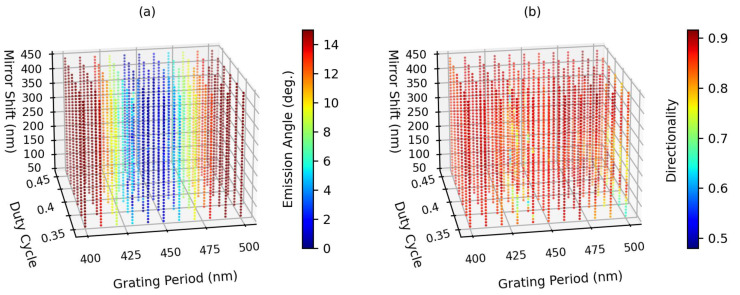
Simulation results for (**a**) the emission angle and (**b**) the directionality in the case of 150 nm hole depth at 1310 nm wavelength. An emission angle <1 degree and a directionality >0.90 simultaneously occur when the grating period is approximately 460 nm, and the duty cycle is approximately 0.39.

We sweep the grating period from 400 nm to 500 nm, the duty cycle from 0.35 to 0.45, the Au mirror shift from 50 nm to 450 nm, and the hole depth or pillar height both from 50 nm to 250 nm. Here, we did not opt for commonly used optimization algorithms such as particle swarm optimization, in order to avoid the problem of being trapped in local extrema and to gain a better understanding of the influence of various parameters on the grating coupler. The relative coupling efficiency by integrating the upward power within the acceptance angle of the optical fiber is calculated, and since the numerical aperture of the SMF optical fiber is set to 0.12, making an acceptance angle of 6.9 degrees, only the upward power within this 6.9 degree would be considered in the calculation. Figure 3 shows the calculated relative coupling efficiencies for various grating periods, duty cycles, Au mirror shifts, and pillar heights. Note that the calculated relative coupling efficiencies in all cases were normalized with respect to the maximum relative coupling efficiency. The highest relative coupling efficiency was found to occur in the case of a 150 nm hole depth (see Figure 3c). To further examine the output emission angle and directionality, we compute the far-field angle of the maximum power detected by the upward power monitor, and the ratio of this upward power to the summation of the upward and downward powers. The emission angle and directionality in the case of 150 nm hole depth are shown in Figure 4. It shows that a minimum emission angle of <1 degree (see Figure 4a) and a maximum directionality of >0.90 (see Figure 4b) can be achieved when the grating period is 460 nm, and the duty cycle is 0.39. Note that with a smaller parameter sweeping step and a finer simulation mesh, an emission angle closer to zero can be obtained. However, the amount of computation will increase dramatically, so we accept this < 1-degree accuracy in evaluating the design of the 2D grating coupler in the following.

## 3. Full Structure Simulation

**Figure 5:**
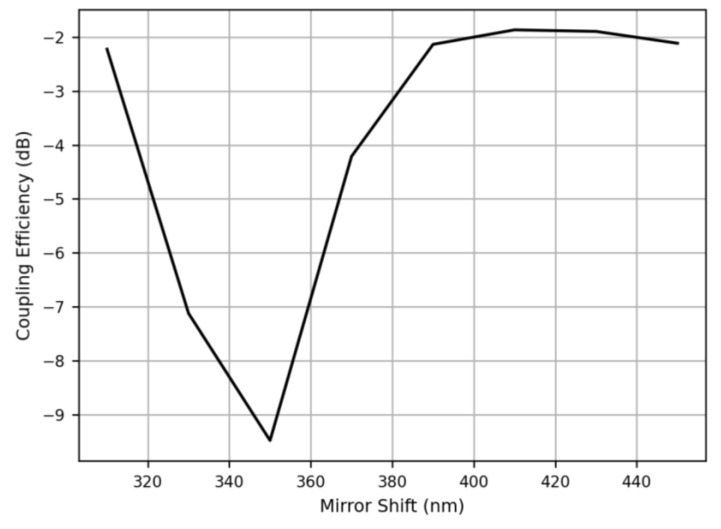
The coupling efficiency simulated as a function of corner mirror shift at 1310 nm wavelength.

Based on the results obtained by simulating the unit-cell structure, we then select the optimal parameters for further simulating the full structure of the 2D grating coupler, including the 2D grating, the two tapered Si waveguides, and the two corner mirrors. For these full structure simulations, we configure the 2D grating coupler as an optical receiver where the fundamental mode of the optical fiber with two orthogonal polarizations is launched and propagates to the 2D grating coupler. The standard SMF-28 optical fiber structure is utilized to generate the precise fundamental mode as an input source, and hence, the mode-matching conditions are also considered in the calculation of coupling efficiency. Two mode power monitors are respectively placed at the two ends of the two tapered Si waveguides. Then, the corner mirror shift is fine-tuned to obtain the highest coupling efficiency. We sweep the corner mirror shift, which is defined as the spacing between the end of the grating pattern and the beginning of the corner mirror, from 310 nm to 450 nm. The coupling efficiency versus the corner mirror shift at 1310 nm wavelength is shown in Figure 5, which indicates that the optimized shift is approximately 410 nm.

**Figure 6:**
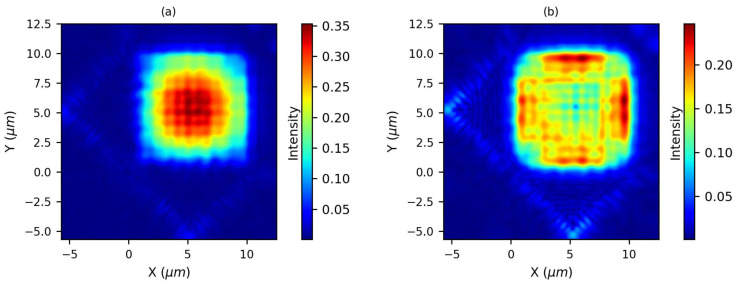
The optical intensity profile (**a**) for 410 nm corner mirror shift and (**b**) for 350 nm corner mirror shift. The Gaussian-like profile of the intensity distribution is essential to achieving high coupling efficiency in optical systems.

In order to investigate the impact caused by the corner mirror shift, the grating coupler is employed as a transmitter, and the input waveguide mode source is placed in the Si waveguide. The output light distribution from the grating coupler is then monitored to examine the effect of the mirror shift. The optical intensity profiles for two different shifts, i.e., 410 nm and 350 nm, are shown in Figure 6a,b, respectively.

The optical intensity profile in Figure 6a, which is obtained at the maximum coupling efficiency due to the formation of the standing wave pattern appropriately matching the 2D grating pattern [17], shows that the optical field distribution is Gaussian-like and nicely confined in the grating region. In contrast, the optical intensity profile in Figure 6b shows that the optical field distribution is exponential-like and undesired for mode matching. Thus, the corner mirror shift of 410 nm is selected in subsequent simulations.

## 4. Results and Discussion

**Figure 7:**
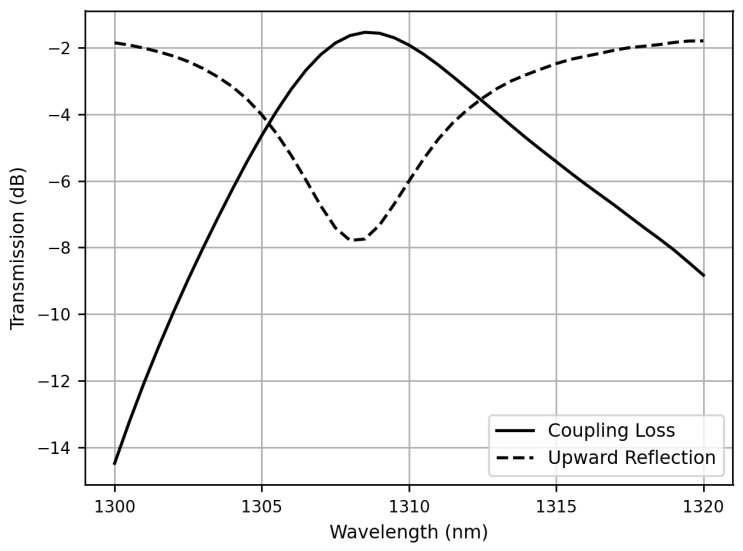
Spectra of coupling efficiency and upward reflection when the 2D grating coupler acts as an optical receiver. The maximum coupling efficiency is −1.53 dB and the upward reflection is −7.7 dB at 1308 nm.

We have obtained the optimized structural parameters and simulated the entire structure as a receiver. In Figure 7, the spectra of coupling efficiency and upward reflection are shown. At the wavelength of 1308 nm, the coupling efficiency reaches its maximum and is found to be −1.53 dB, the highest coupling efficiency among the 2D grating couplers without a bottom mirror reported in the literature. The slight deviation of the wavelength from 1310 nm here is caused by the sweep step of the parameters. By using a finer scanning interval, a more precise wavelength can be obtained. The corresponding upward reflection refers to the light that is reflected by the grating coupler back towards the input fiber, and the minimum reflection is measured to be −7.7 dB at the wavelength of 1308 nm. The spectral bandwidth of coupling is relatively narrow and is approximately 5 nm at 1-dB loss. This narrow bandwidth is due to the necessary constraint of forming the desired Gaussian-like standing wave pattern. Although the spectral bandwidth of coupling is relatively narrow, it remains a feasible option for applications where precise wavelength control is needed. Moreover, owing to the advancement of modern laser products where highly accurate control over the laser wavelength within a few nanometers can be achieved nowadays, the narrow bandwidth issue shown in Figure 7 can be mitigated. On the other hand, the shift of the optimal coupling wavelength due to fabrication process variation may occur. However, using relevant techniques demonstrated in previous studies over micro-rings [18,19], fine-tuning the optimal coupling wavelength to match the laser wavelength is still possible. Therefore, despite the inherent limitations imposed by resonance, this proposed structure can still be implemented for various applications.

**Figure 8:**
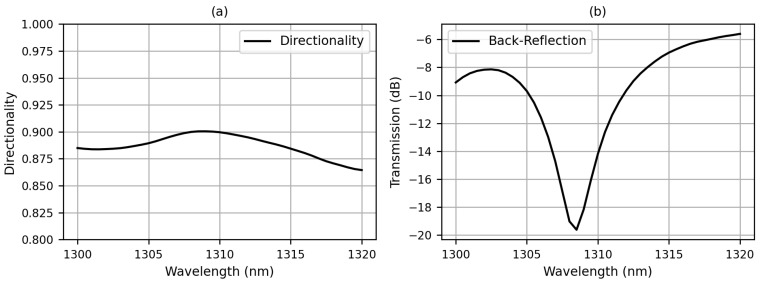
Spectra of (**a**) directionality and (**b**) back-reflection when the 2D grating coupler acts as an optical transmitter. The directionality is >0.90 and the back-reflection is −19.6 dB at 1308 nm.

In addition, we also perform simulations for the case when the 2D grating coupler is configured as an optical transmitter. The spectra of directionality and back-reflection are shown in Figure 8. The directionality is found to be between 0.86 and 0.90 at wavelengths from 1300 nm to 1320 nm, while the corresponding back-reflection shows a dip of −19.6 dB at 1308 nm.

**Figure 9:**
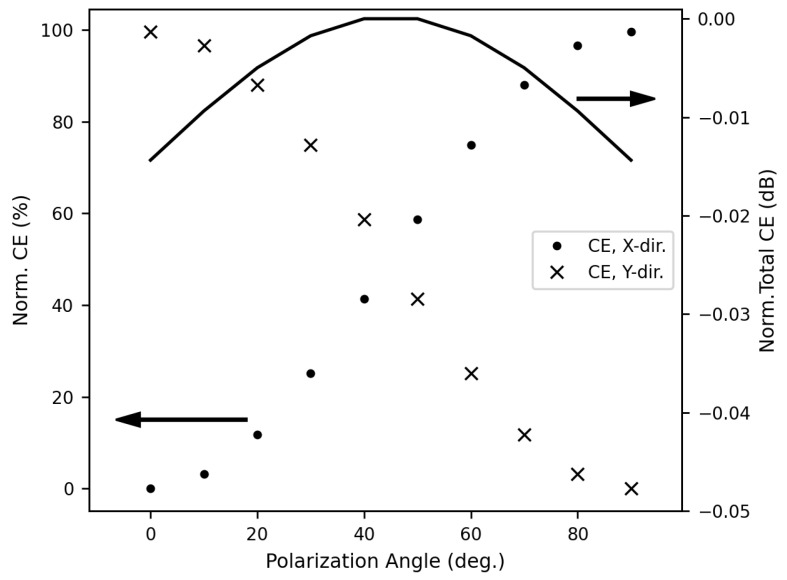
Normalized coupling efficiencies versus polarization angle at 1308 nm wavelength. Circles and crosses represent the coupling efficiencies for coupling to the x-direction and the y-direction (indicated by the left arrow), respectively, and the solid line is the total coupling efficiency (indicated by the right arrow). The resultant PDL is below 0.015 dB.

**Figure 10:**
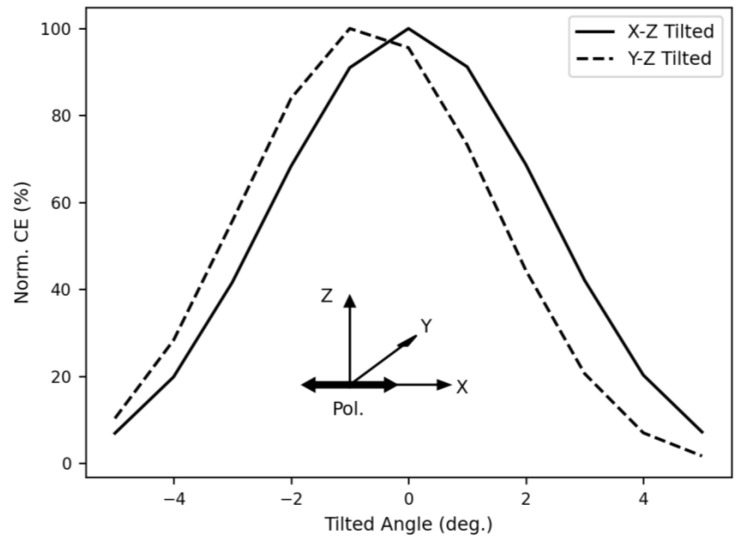
Normalized coupling efficiency versus tilt angle at 1308 nm wavelength. The polarization of the input source is in the x-direction, and the source is tilted either on the x-z plane or the y-z plane.

Next, we calculate the PDL using a Gaussian mode source vertically incident on the 2D grating coupler with its polarization rotated to a different angle in each simulation. The two-mode power monitors are used to calculate the transmitted powers in the x and y directions, respectively. The results for PDL calculations are shown in Figure 9. As expected, the PDL is very low and can be as small as 0.015 dB because the 2D grating coupler is invariant under a 90° rotation along the optical fiber axis. In addition, the fiber tilt angle tolerance is also calculated. In this case, a Gaussian mode source with polarization in the x-direction is incident on the 2D grating coupler with the incidence angle tilted on either the x-z or y-z plane, respectively. Again, the two-mode power monitors are used to calculate the transmitted powers in the x and y directions, respectively. In Figure 10, the highest coupling efficiency occurs when there is no tilt on the x-z plane, and where there is a slight tilt of approximately 1 degree on the y-z plane. As explained in the previous paragraph, this 1 degree is due to the limited resolution of simulating and can be eliminated by a smaller sweep step and a finer simulation mesh. The resultant tilt tolerance, defined by a 3-dB drop in coupling efficiency, is ± 2.3 degrees.

Table 1 summarizes the performances of the 2D grating couplers reported in the literature in recent years. As the table shows, our 2D grating coupler has the highest coupling efficiency compared to the 2D grating couplers without a bottom mirror. Its coupling efficiency can even be comparable to or better than the 2D grating couplers with a bottom mirror. Moreover, important properties such as being completely vertical and polarization independent can be simultaneously achieved, making it a promising candidate for fiber-to-chip as well as chip-to-chip optical couplings.

## 5. Conclusions

An efficient 2D grating coupler as an optical receiver is designed, where desired properties such as being completely vertical and polarization-independent can be achieved. This is made possible by utilizing the second-order diffraction for the formation of the standing wave pattern appropriately matching the 2D grating pattern [17], through corner mirrors respectively placed at the two orthogonal ends of the 2D grating coupler and a 2D grating pattern consisting of arrays of holes or pillars in a square-lattice configuration. The maximum coupling efficiency from a standard single-mode fiber to the Si waveguide is only −1.53 dB at 1308 nm, which is the highest coupling efficiency ever reported for a 2D grating coupler without a bottom mirror. We also show that this 2D grating coupler can be designed with minimal computational resources by recognizing symmetries for simplification. Finally, it is worth noting that our design can be fabricated by standard 248 nm DUV lithography and there is no bottom mirror or intricate structures that may cause process complications.

## Figures and Tables

**Table 1:** Comparison of 2D grating couplers.

Ref.	Year	Band	Fiber Angle	Apodization	Bottom Mirror	Coupling Efficiency	
						Sim	Exp
[7]	2018	C	12	-	Y	−1.4	−1.8
[20]	2019	C	12	-	-	−3.46	−4.2
[9]	2019	C	0	Y	-	−2.4	−2.6
[21]	2020	O	10	-	Y	−1.73	−2.37
[22]	2021	C	0	Y	-	−1.87	−3.1
This work		O	0	-	-	−1.53	-

## Data Availability

Not applicable.
